# The Level of Awareness of Keratoconus Among the General Population in Hail Region, Saudi Arabia

**DOI:** 10.7759/cureus.50026

**Published:** 2023-12-06

**Authors:** Zaki A Alshammari, Abrar Ali, Layan K Alshammari, Othman M Alassaf, Ali Yahya A Alshehri, Reem AlSarhan, Basmah Alanazi

**Affiliations:** 1 Department of Ophthalmology, University of Hail College of Medicine, Hail, SAU; 2 Department of Ophthalmology, King Khalid Hospital, Hail, SAU; 3 Department of Ophthalmology, King Khalid University, Abha, SAU; 4 Clinical Sciences Department, College of Medicine, Princess Nourah Bint Abdulrahman University, Riyadh, SAU

**Keywords:** hail, saudi arabia, knowledge, awareness, cone-shaped cornea, conical cornea, kcn, kc, keratoconus

## Abstract

Background

Keratoconus (KC) is a non-inflammatory corneal disease with an early onset in adulthood, leading to a reduction in visual acuity. This study aims to evaluate the level of awareness of keratoconus among the general population in the Hail region of Saudi Arabia.

Methodology

Data were collected through a pre-designed and pre-validated online questionnaire (Appendix) distributed via social media platforms. The questionnaire was divided into two sections. The first section included demographic profiles, while the second section inquired about knowledge and awareness regarding Keratoconus. The collected data was reviewed, coded, and inputted into IBM Corp. Released 2013. IBM SPSS Statistics for Windows, Version 22.0. Armonk, NY: IBM Corp. Statistical analyses were performed using the Pearson Chi-Square test, with statistical significance set at p<0.05.

Results

The total number of respondents was 550, among whom 40% were males and 60% were females. 79.6% of the participants were in the age range of 18-30 years. The level of education and a positive family history of KC showed significant associations with the level of knowledge about KC (p<0.05). The age group had a non-significant association (p=0.059), while gender had a significant association with the level of knowledge about keratoconus (p<0.05).

Conclusion

In conclusion, the overall awareness regarding KC progression, interventions, and the consequences of eye rubbing was limited among the participants. Specific efforts are crucial to enhance public awareness and understanding of KC, ensuring a more informed and proactive approach to eye health within the community.

## Introduction

Keratoconus (KC) is a non-inflammatory corneal disease that affects both genders and has an early onset in adulthood [1]. It manifests as bilateral progressive steepening and thinning of the central or paracentral cornea; the thinnest corneal location is typically intertemporal. It is linked to a reduction in visual acuity, most notably myopia and irregular astigmatism [2]. The etiology is still unclear, but is associated with risk factors such as eye rubbing [3], sun exposure, atopy, genetic and environmental factors [4]. There is a notable correlation between increasing age and the severity of keratoconus symptoms [5]. In the Middle East, the prevalence of KC is up to 5%, and geographically, the mountainous regions have the highest rate of KC patients [1,6]. KC is classified into four stages: subclinical, early, moderate, and severe, according to clinical presentation and topography. In the subclinical stage, there is only suspicious keratoconus on topography with a normal slit lamp examination and visual acuity. In the early stage, mild differences in refractive error and reduction in visual acuity can be present, along with mild, localized corneal steepening and thinning, increasing keratometric differences between the inferior and superior cornea, and increases in corneal aberrations, the scissor reflex, and Charles Louis' oil droplet reflex [2]. Slit-lamp examination can detect advanced keratoconus, which includes the classic clinical signs Munson's sign, Vogt's striae, and Fleischer ring, as well as severe corneal thinning and steepening (>55 D) and corneal scarring [2,7]. Tomographic imaging is used in the diagnosis and screening of KC [8]. The diagnosis of keratoconus needs to be made early because good outcomes are associated with early intervention [9]. KC treatment in early diagnosis includes glasses and contact lenses, as well as surgical intervention such as intracorneal ring segment (ICRS) implantation and corneal crosslinking (CXL), whereas in late diagnosis, it is not possible to practice, and patients are left with only penetrating keratoplasty (PK) or deep anterior lamellar keratoplasty (DALK) [9,10]. This study is being conducted to assess the level of awareness regarding KC among the general population in the Hail region of Saudi Arabia.

## Materials and methods

This is a cross-sectional analytical study that was conducted from July 2023 to November 2023. The study population consisted of adult males and females residing in Hail City, Saudi Arabia. The inclusion criteria comprised individuals of both genders aged 18 years and older who reside in Hail. Exclusion criteria included males and females under 18 years old and individuals living in regions other than Hail. The calculated sample size was based on the latest census in the Hail region (746,406). Using the Roasoft calculator with a 5% margin of error and a 95% confidence interval, the estimated calculated sample size was 385 participants. However, the final sample size was 550 after data collection.

Data were collected through a pre-designed and pre-validated online questionnaire from a prior study conducted in Jeddah, Saudi Arabia [11]. The questionnaire was distributed using a self-administered Google form through a WhatsApp broadcast messaging app (Meta Platforms, Inc., Menlo Park, California, United States) that contained the questionnaire, research objectives, and study rationale. The questionnaire was translated into Arabic and then back into English. The validity of the questionnaire was tested through a pilot study, and the data from the pilot study were excluded from the main study. 

The questionnaire was preceded by informed consent, which ensured the confidentiality of the data. The questionnaire consisted of 24 questions and was divided into two sections. The first section inquired about the socio-demographic profile. This section was utilized to confirm the inclusion criteria of the study participants, including the presence of an underlying allergy or ophthalmic condition, a history of ophthalmic surgery, or a family history of KC, while the second section used 10 items to assess the knowledge and awareness regarding KC and two items to assess the frequency of participants eye rubbing habits and their reasoning for the mentioned habit. This study has been reviewed and approved by the Research Ethics Committee (REC) at the University of Hail.

Data analysis

The data collected were reviewed, coded, and inputted into IBM Corp. Released 2013. IBM SPSS Statistics for Windows, Version 22.0. Armonk, NY: IBM Corp. after extraction. Statistical analyses were performed using the Pearson Chi-Square test, with statistical significance set at p<0.05. For knowledge and awareness questions, each correct response was assigned a score of one point, and the total score was calculated. Poor awareness was defined as a score below 5 points, moderate awareness fell within the range of 5 to 7 points, and a score of at least 8 points denoted good awareness. Descriptive analysis was performed on all variables, including participants' biographical data, family history of KC, medical history of ophthalmic conditions, and the frequency of eye rubbing, based on frequency and percent distribution. Frequency tables and graphs were used to assess participants' awareness levels regarding KC. Cross-tabulation was used to determine the distribution of participants' awareness levels based on their personal data and practices.

## Results

The study questionnaire was completed by 550 individuals. Table 1 shows that 79.6% of the participants were in the age range of 18-30 years, while the 31-40 age group constituted 12% of the study population. Furthermore, among the study population, 60.2% were females, and 67.6% held a university degree, while high school graduates accounted for 29.5% of the participants. About 30.5% reported having some form of allergy, with eye atopy being the most frequently reported (16.9%), followed by skin allergies (11.3%) and asthma (8.2%). 47.5% of the participants had an underlying eye condition, with refractive errors being the most common eye disorder (34.4%), followed by chronic dry eyes (17.8%). Of the participants, 34 reported having a diagnosis of KC, while 65 participants reported a family history of KC. Fifty-nine participants reported undergoing refractive surgery, while 14 had undergone cataract removal surgery. Moreover, 36.5% of the participants had previously heard about KC, with the most commonly reported sources of information being the internet and social media (14%), followed by lectures and reading (13.5%), and friends and family (10.1%).

**Table 1:** Demographic data of the study’s participants

		Count	Percentage
Age group	18-30	438	79.6%
	31-40	66	12.0%
	41-50	28	5.1%
	51-60	13	2.4%
	Older than 60	5	0.9%
Sex	Male	219	39.8%
	Female	331	60.2%
Level of education	Uneducated	1	0.2%
	Primary school	3	0.5%
	Middle school	13	2.4%
	High school	161	29.3%
	University Degree	372	67.6%
Had a form of allergy	Yes	168	30.5%
	No	382	69.5%
If yes, what form of allergy	Eye allergy	93	16.9%
	Chest allergy	45	8.2%
	Skin allergy	62	11.3%
	GIT allergy	12	2.2%
	Allergic Rhinitis	9	1.6%
Diagnosed with an ophthalmic condition	Yes	261	47.5%
	No	289	52.5%
If yes, what is the condition	Refractive error	189	34.4%
	Dry eyes	98	17.8%
	Cataract	8	1.5%
	Glaucoma	5	0.9%
	Diabetic retinopathy	4	0.7%
	Keratoconus	34	6.2%
	Amblyopia	3	0.5%
Had an ophthalmic surgery	Refractive surgery	59	10.7%
	Cataract removal surgery	14	2.5%
	Glaucoma surgery	6	1.1%
	Cornea cross-linking	2	0.3%
	Retinal laser and‎/or Anti-VEFG injections	1	0.18%
	Corneal transplant	2	0.3%
Experienced a progressive decline in visual acuity	Yes	156	28.4%
	No	239	43.5%
	Maybe	155	28.2%
Family history of Keratoconus	Yes	65	11.8%
	No	485	88.2%
Have heard of Keratoconus	Yes	201	36.5%
	No	349	63.5%
What is your source of information	Lectures‎/reading	74	13.5%
	Internet‎/social media	77	14.0%
	Family‎/friends	56	10.1%
	Diagnosed Relative	26	4.7%
	Doctors‎	50	9.1%
	No specific source	106	19.3%

Table 2 reports participants' answers to the knowledge and awareness items used in the current study's questionnaire. It reveals that only 21.4% of the participants correctly reported that KC is characterized by the thinning of the cornea. Regarding the hereditary aspect of KC, 32.9% answered correctly that it has a hereditary element. Furthermore, 44.2% correctly reported that KC is related to allergies and eye rubbing. When asked about the adverse effect of KC on visual acuity, the majority of participants answered correctly (53.3%). In terms of the progression of KC to blindness, 19.8% answered correctly. Regarding the possibility of surgical intervention for advanced KC, 44.7% answered correctly. When it comes to wearing prescription glasses, 17.6% answered correctly that it may not halt the progression of the disease. When asked if KC necessitates frequent doctor evaluations and follow-ups, most of the participants answered correctly (54.4%). Moreover, half of the participants (50.4%) correctly reported that eye rubbing may lead to KC, while only 5.5% stated that it was a safe habit. Among the study subjects, only 4.4% believed that no treatment was available for KC, while 15.5% mentioned having the habit of frequently rubbing their eyes. The most common reasons for eye rubbing were itchiness, followed by stress and headaches (37.8%).

**Table 2:** Awareness and perception of keratoconus among study participants

		Count	Percentage
Keratoconus may be described as	Autoimmune disease	14	2.5%
	Inflammation in the cornea	58	10.5%
	Increase in the thickness of the cornea	81	14.7%
	I do not know	201	36.5%
	Decrease in the thickness and coning of the cornea	196	35.6%
is Keratoconus hereditary?	Yes	181	32.9%
	No	92	16.7%
	I do not know	277	50.4%
Is there a relationship between Keratoconus and eye rubbing or allergy?	Yes	243	44.2%
	No	79	14.4%
	I do not know	228	41.5%
Can Keratoconus affect visual acuity?	Yes	293	53.3%
	No	35	6.4%
	I do not know	222	40.4%
Can Keratoconus progress to blindness?	Yes	126	22.9%
	No	109	19.8%
	I do not know	315	57.3%
Does advanced Keratoconus require a surgical intervention?	Yes	246	44.7%
	No	46	8.4%
	I do not know	258	46.9%
Does wearing prescription glasses halts the progression of Keratoconus?	Yes	135	24.5%
	No	97	17.6%
	I do not know	318	57.8%
Does Keratoconus necessitate frequent Doctor evaluation and follow ups?	Yes	299	54.4%
	No	32	5.8%
	I do not know	219	39.8%
Frequent eye rubbing may be considered?	A habit that may lead to Keratoconus	277	50.4%
	It is a safe habit	30	5.5%
	I do not know	243	44.2%
What are the treatment modalities for keratoconus?	Prescription glasses	137	24.9%
	Medical lenses	125	22.7%
	Corneal transplant	157	28.5%
	Corneal cross-linking	146	26.5%
	Eye drops	120	21.8%
	There is no treatment	24	4.4%
	I do not know	230	41.8%
How Frequent do you rub your eyes?	Always	85	15.5%
	Sometimes	281	51.1%
	Rarely	184	33.5%
What is the Reason for the rubbing?	Stress or headache	208	37.8%
	Itchiness	413	75.1%
	Allergy	170	30.9%
	Dryness or irritation	10	1.8%

**Figure 1:**
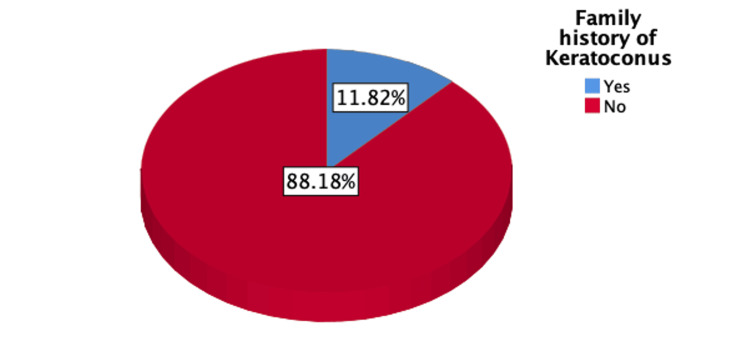
Percentage distribution of the participants according to having a family history of Keratoconus

**Figure 2:**
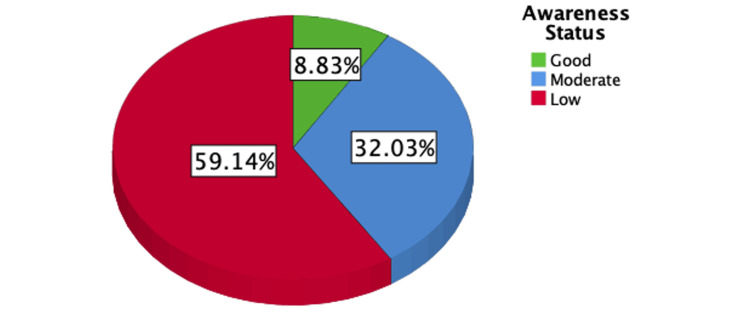
The percentage distribution of the participants according to their knowledge level about Keratoconus.

Figure 1 illustrates that 11.8% of the participants had a family history of KC, while Figure 2 illustrates the distribution of poor, moderate, and good knowledge levels regarding Keratoconus among the participants, with percentages of 59.14%, 32.02%, and 8.8%, respectively. 

Table 3 shows that the level of education and having a family history of keratoconus had significant associations with the level of knowledge about keratoconus (p<0.05). The age group had a non-significant association (p=0.059), while sex had a significant association with the level of knowledge about keratoconus (p<0.05). A significant relationship was found between participants' knowledge level about KC and having a form of allergy (p=0.00). Moreover, being diagnosed with an ophthalmic condition also had a significant relationship with the level of knowledge (p<0.00). However, other characteristics, such as undergoing ophthalmic surgery and the frequency of eye rubbing, had a non-significant association.

**Table 3:** There is an association between Keratoconus awareness and participants' demographics, allergy or eye condition history, ophthalmic surgery, family Keratoconus history, and eye rubbing frequency.

		Awareness Status						
		Good		Moderate		Low		
		No.	N %	No.	N %	No.	N %	P-value
Age group	18-30	39	10.2%	134	34.9%	211	54.9%	0.059
	31-40	3	5.0%	13	21.7%	44	73.3%	
	41-50	1	3.6%	7	25.0%	20	71.4%	
	51-60	0	0.0%	1	10.0%	9	90.0%	
	Older than 60	0	0.0%	1	20.0%	4	80.0%	
Sex	Male	11	5.8%	40	20.9%	140	73.3%	<0.001
	Female	32	10.8%	116	39.2%	148	50.0%	
Level of education	Uneducated	0	0.0%	0	0.0%	1	100.0%	<0.001
	Primary school	0	0.0%	0	0.0%	3	100.0%	
	Middle school	0	0.0%	1	9.1%	10	90.9%	
	High school	8	5.4%	29	19.5%	112	75.2%	
	University degree	35	10.8%	126	39.0%	162	50.2%	
Had a form of allergy	Yes	16	10.9%	65	44.2%	66	44.9%	<0.001
	No	27	7.9%	91	26.8%	222	65.3%	
Diagnosed with an ophthalmic condition	Yes	33	14.6%	94	41.6%	99	43.8%	<0.001
	No	10	3.8%	62	23.8%	189	72.4%	
Had an ophthalmic surgery	Refractive surgery	4	7.5%	20	37.7%	29	54.7%	0.451
	Cataract removal surgery	3	21.4%	3	21.4%	8	57.1%	
	Glaucoma surgery	1	20.0%	1	20.0%	3	60.0%	
	Cornea cross-linking	0	0.0%	1	100.0%	0	0.0%	
	Retinal laser and‎/or Anti-VEFG injections	0	0.0%	0	0.0%	1	100.0%	
	Corneal transplant	1	50.0%	1	50.0%	0	0.0%	
Family history of Keratoconus	Yes	10	18.5%	19	35.2%	25	46.3%	0.015
	No	33	7.6%	137	31.6%	263	60.7%	
Eye rubbing frequency	Always	5	6.5%	19	24.7%	53	68.8%	0.403
	Sometimes	23	9.0%	83	32.4%	150	58.6%	
	Rarely	15	9.7%	54	35.1%	85	55.2%	

## Discussion

The importance of awareness and knowledge about KC plays a significant role in the emergence and prognosis of the disease. A study conducted in Saudi Arabia shows that only 27.2% (n = 113) provided a correct definition of KC [12]. According to another study in the Aseer region, it was found that 23.6% of participants aged 30-39 years had a good awareness level compared to 9.1% of older participants, with recorded statistical significance (P = 0.049) [13]. Given the fluctuation in awareness percentages, it is essential to educate society about the nature of the disease, its risk factors, and available treatment measures.

In a nationwide study conducted in 2021, a poor knowledge score of 67.5% was reported regarding KC despite its high prevalence in Saudi Arabia compared to other Middle Eastern countries [12]. In this study, 550 participants from the Hail region in Saudi Arabia, including 60.2% females and 39.8% males, provided data. The calculated levels of poor, moderate, and good knowledge regarding keratoconus among the participants were 59.14%, 32.02%, and 8.8%, respectively.

Of the participants, 32.9% (n=181) acknowledged the hereditary nature of keratoconus, and 11.8% (n=65) had a family history of the disease. Research linking genetics to KC suggests that family history plays a significant role, in line with previous reports that genetics may influence keratoconus presentations [14-16].

In 2020, a study reported that 73% of keratoconus patients admitted to aggressively squeezing their eyes [13]. This analysis supports the theory that there is a relationship between KC and the habit of eye rubbing, which was appreciated by nearly half of our respondents (44.2%). In terms of the complications of keratoconus, respondents' insufficient knowledge may have been caused by important misunderstandings. Only 19.8% correctly identified the unlikelihood of KC progressing to blindness. Almost a fifth of the participants (19.3%) cited no specific source as their primary source of knowledge. Other sources of information came from the internet and social media (14.0%), lectures and reading (13.5%), followed by close friends and relatives (10.1%). Healthcare professionals were ranked fifth as a source of information. Young age, high education, having a disease-causing visual impairments, and using a doctor as the primary information source were the most significant predictors of public awareness.

Regarding the treatment of KC, 44.7% correctly answered about the possibility of surgical intervention for advanced keratoconus. When it comes to wearing prescription glasses, only 17.6% correctly answered that it may not halt the progression of the disease.

Education emerged as a determinant influencing participants' knowledge about KC, consistent with another study in the Aseer region of Saudi Arabia [17]. However, it is common for higher education to correlate with increased health awareness [18,19]. This underscores the need for targeted educational campaigns, particularly for individuals with limited educational backgrounds, to enhance their comprehension of KC. Interestingly, age displayed no significant association with KC knowledge, contrasting studies conducted in various regions of Saudi Arabia [12,17,20]. A study by Al-Amri et al. revealed that younger individuals exhibited a poorer understanding of KC [21].

Moreover, family history emerged as a substantial risk factor for KC [15,22], significantly influencing participants' knowledge. While some studies in Saudi Arabia found family history nonsignificant [12,17], others identified it as significant [20,23], potentially due to variations in knowledge levels and methodologies. This suggests that individuals with a family history of KC engage in information-seeking behaviors, gaining knowledge through research and ophthalmology visits. This correlation emphasizes the role of familial background in shaping KC awareness, enabling healthcare professionals to target specific demographics for educational interventions and awareness campaigns. Such efforts may lead to early detection and improved management within affected families.

Additionally, gender exhibited a significant association with KC knowledge, contrasting previous studies that found it nonsignificant [12,17,23]. This underscores the need for gender-specific educational campaigns to bridge the knowledge gap effectively. Concerning allergies, particularly systemic and ocular allergies, these conditions were key risk factors associated with KC pathogenesis [24].

Participants with allergies or previous ocular conditions displayed higher KC knowledge, likely due to their regular interactions with healthcare providers. It is well known that individuals with pre-existing health conditions tend to possess increased awareness. Therefore, integrating KC-related information into broader healthcare discussions, especially for patients with allergies or other ocular conditions, is crucial.

Although eye rubbing constitutes a risk factor for KC [3], this study found no significant association between ophthalmic surgery, eye rubbing frequency, and KC knowledge. This implies that these factors might not significantly influence participants' awareness of KC. This result is consistent with previous studies in the Aseer region and Medina in Saudi Arabia [17,23], which also found no significant association despite a high prevalence of eye rubbing. Nevertheless, further studies are needed to investigate this non-association. However, the study had certain limitations, notably its non-probability nature and the restriction to the Hail region of Saudi Arabia, which might limit the generalizability of the findings to a broader population.

## Conclusions

In conclusion, the study underscores a notable gap in knowledge about keratoconus, particularly among participants in the 18-30 age group. Despite a significant prevalence of family history related to KC, only a small fraction exhibited a correct understanding of crucial aspects of the condition. The overall awareness regarding keratoconus progression, interventions, and the consequences of eye rubbing was limited among the participants. The identified associations between knowledge level and factors such as education, family history, age, and gender emphasize the necessity for focused educational initiatives. Specific efforts are crucial to enhance public awareness and understanding of keratoconus, ensuring a more informed and proactive approach to eye health within the community.

## Appendices

Questionnaire 

**Table 4:** A list of questionnaire items

Tool 1: Demographic and ophthalmic items used in the study	
Age group	18-30
	31-40
	41-50
	51-60
	Older than 60
Sex	Male
	Female
Level of Education	Uneducated
	Primary school
	Middle school
	High school
	University Degree
Had a form of allergy	Yes
	No
If yes, what form of allergy	
	Eye allergy
	Chest allergy
	Skin allergy
	GIT allergy
	Allergic Rhinitis
Diagnosed with an Ophthalmic condition	Yes
	No
If yes, what is the condition	
	Refractive error
	Dry eyes
	Cataract
	Glaucoma
	Diabetic retinopathy
	Keratoconus
	Amblyopia
Had an Ophthalmic surgery	Refractive surgery
	Cataract removal surgery
	Glaucoma surgery
	Cornea cross-linking
	Retinal laser and‎/or Anti-VEFG injections
	Corneal transplant
Experienced a progressive decline in visual acuity	Yes
	No
	Maybe
Family history of Keratoconus	Yes
	No
Have heard of Keratoconus	Yes
	No
What is your source of information	
	Lectures‎/reading
	Internet‎/Social media
	Family‎/friends
	Diagnosed Relative
	Doctors‎
	No specific source
Tool 2: Awareness and knowledge items used in the study	
Keratoconus may be described as	Autoimmune disease
	Inflammation in the cornea
	Increase in the thickness of the cornea
	I do not know
	Decrease in the thickness and coning of the cornea
is Keratoconus hereditary?	Yes
	No
	I do not Know
Is there a relationship between Keratoconus and eye rubbing or allergy?	Yes
	No
	I do not Know
Can Keratoconus affect visual acuity	Yes
	No
	I do not Know
Can Keratoconus progress to blindness?	Yes
	No
	I do not Know
Does advanced Keratoconus require a surgical intervention?	Yes
	No
	I do not Know
Does wearing prescription glasses halts the progression of Keratoconus?	Yes
	No
	I do not Know
Does Keratoconus necessitate frequent Doctor evaluation and follow ups?	Yes
	No
	I do not Know
Frequent eye rubbing may be considered?	A habit that may lead to Keratoconus
	It is a safe habit
	I do not know
What are the treatment modalities for keratoconus?	
	Prescription glasses
	Medical lenses
	Corneal transplant
	Corneal cross-linking
	Eye drops
	There is no treatment
	I do not know
Eye rubbing frequency	Always
	Sometimes
	Rarely
If yes, what is the Reason?	
	Stress or headache
	Itchiness
	Allergy
	Dryness or irritation
